# Mycorrhizal Fungal Inoculation Reshapes Chemotype, Nutritional Status, and Metabolic Signatures to Enhance Bioactivity in *Origanum compactum* Benth.

**DOI:** 10.3390/plants15162518

**Published:** 2026-08-20

**Authors:** Akhallaa Youne Oumnia, Akhallaa Youne Mounia, Ouahmane Kaoutar, Rhouch Said, Bouskout Mohammed, Hicham Kaddouri, Alfeddy Mohamed Najib, Mnasri Bacem, Tounsi Abdessamad, Dounas Hanane, Hina Nazameen, Yaseen Khan, Ouahmane Lahcen

**Affiliations:** 1 Laboratory of Water Sciences, Microbial Biotechnologies and Natural Resources Sustainability, Unit of Microbial Biotechnologies, Agrosciences, and Environment-CNRST Labeled Research Unit N°4, Faculty of Sciences Semlalia, Cadi Ayyad University, P.O. Box 2390, Marrakesh 40000, Moroccomohammed.bouskout@ced.uca.ma (B.M.);; 2 Phytobacteriology Laboratory Plant Protection Research, Unit CRRA Marrakesh National Institute for Agronomical Research Marrakesh, Marrakesh 40000, Morocco; 3 Laboratory of Legumes and Sustainable Agrosystems, Centre of Biotechnology of Borj-Cédria, BP 901, Hammam-Lif 2050, Tunisia; 4 Environmental, Ecological and Agro-Industrial Engineering Laboratory, Faculty of Sciences and Techniques, University Sultan Moulay Slimane, Avenue Mohamed V. BP 591, Beni Mellal 23000, Morocco; 5 Department of Botany, Bacha Khan University, Charsadda 24420, Pakistan; hinanazameen@gmail.com; 6 Center for Eco-Environment Restoration Engineering of Hainan Province, School of Ecology, Hainan University, Haikou 570228, China

**Keywords:** *Origanum compactum*, mycorrhizae, essential oils, phytochemical composition, antioxidant activity, antibacterial activity

## Abstract

Arbuscular mycorrhizal fungi (AMF) establish a reciprocal interaction with plant roots, enhancing nutrient acquisition, stress tolerance, and the production of bioactive metabolites. These symbiotic fungi represent a sustainable alternative to chemical fertilizers to improve the quality and yield of medicinal and aromatic plants such as Oregano (*Origanum compactum*). In this study, we investigated the effects of mycorrhizal inoculation on the chemical composition, nutritional profile, and biological activities of Oregano cultivated under greenhouse conditions. Compared with non-mycorrhizal plants, mycorrhizal-inoculated plants showed approximately 33% higher protein content and 28% higher total sugar content, while lipid concentration decreased slightly by about 7%. Mycorrhizal inoculation also promoted the accumulation of secondary metabolites, resulting in increased concentrations of total polyphenol and flavonoid contents by approximately 33% and 25%, respectively. These compositional changes were associated with markedly enhanced antioxidant capacity, exceeding that of the reference antioxidant, as well as improved antibacterial activity, characterized by larger inhibition zones and lower minimum inhibitory concentrations against tested pathogens. Overall, mycorrhizal fungal inoculation reshaped the chemical composition and the major bioactive compounds of Oregano, thereby enhancing its nutritional and antimicrobial potential. These findings highlight the potential of AMF-based cultivation strategies to improve the phytochemical quality and medicinal potential of Oregano while supporting sustainable agricultural production.

## 1. Introduction

Oregano *(Origanum compactum* benth.) is a medicinal plant belonging to the *Lamiaceae* family [1], which includes 250 genera and approximately 7000 species [2]. Compact-flowered Oregano is endemic to Morocco and the Iberian Peninsula [3,4] and is among the 978 taxa representing over half of North Africa’s endemic flora [5]. This species is widely distributed across northern and central Morocco and occurs in southern Spain, particularly in Andalusia [6]. Morphologically, Oregano displays pubescent stems, ovoid hairy leaves, inflorescences formed by clusters of short violet flowers, rigid lanceolate floral bracts, and a calyx with five triangular teeth [7]. It thrives in Moroccan matorrals, forests, bushes and scrublands, favoring well-drained, dry soils—either limestone or rocky—at altitudes of 700 m above sea level [8]. As the foremost aromatic herb in Moroccan gastronomy, Oregano is prized for its intense flavor and versatility in diverse dishes [9,10]. It is also revered as a “panacea” in traditional lore [11], with flowers, leaves, and stems commonly prepared as decoctions or infusions [12]. These preparations are used to alleviate gastrointestinal discomfort, gastric acidity, and bronchopulmonary disorders [13]; manage diabetes and cardiovascular issues [14]; mitigate respiratory complications, act as a gastric stimulant and febrifuge [15]; and stimulate appetite [16]. In addition, diverse studies have determined its cytotoxic and antitumor effects [17], including activity against breast cancer [18], lung and hepatic cancer [19], rhabdomyosarcoma and kidney cancer [20]. Its anti-inflammatory properties have also been established [17]. Dietary supplements formulated as Oregano capsules are increasingly developed to harness and deliver these therapeutic properties [21].

Essential oils, distilled from medicinal plants, represent a rich source of bioactive compounds exhibiting diverse therapeutic properties [22]. The essential oil of compact-flowered *O. compactum* exhibits a broad spectrum of biological activities, including antidiabetic [23], radioprotective [24], anti-urease and anticholinesterase properties [2]. This essential oil has also been shown to induce a significant increase in lymphocyte micronuclei in human blood samples [25]. Furthermore, it demonstrates insecticidal activity, which supports its potential use in agricultural applications [26].

Mycorrhiza refers to a symbiotic association between fungal hyphae and plant roots, which alters root morphology in a fungus-specific manner [27]. Mycorrhizae are broadly classified into ectomycorrhizal and endomycorrhizal associations [28]. Arbuscular mycorrhizal fungi (AMF) represent a major group of endomycorrhizae and occur in more than 80% of terrestrial plants, including members of the Lamiaceae family [29]. They colonize root cortical cells and form intracellular arbuscules, vesicles, and hyphal coils that mediate nutrient exchange [30]. By extending beyond the root depletion zone, AMF hyphae increase the effective soil volume explored by the plant and can improve the acquisition of water and mineral nutrients, including phosphorus, potassium, magnesium, calcium, copper, zinc, silicon, and sulfur [31,32,33,34,35,36,37,38]. This improved nutrient acquisition may promote plant growth, photosynthetic performance, and tolerance to environmental constraints such as salt stress, drought, and disease [38,39,40]. AMF can also modify the mobility and accumulation of trace elements and heavy metals; however, these effects vary according to the fungal taxon, plant species, soil properties, and metal involved. Mycorrhizal associations may either immobilize potentially toxic elements in the rhizosphere and fungal structures or enhance their uptake by the host plant.

Because of their capacity to improve plant nutrition, growth, and stress tolerance, AMF are increasingly used as biostimulants and biofertilizers in sustainable agriculture, potentially reducing reliance on synthetic fertilizers and other chemical inputs [41]. AMF themselves produce secondary metabolites with potential therapeutic applications against a range of diseases [42,43]. Nevertheless, their effectiveness is influenced by agronomic and environmental factors, including soil fertility, phosphorus availability, tillage, pesticide application, heavy-metal contamination, and host-plant identity [44,45,46,47,48].

Higher plants remain an underexplored source of medicinally valuable bioactive compounds, as only a small proportion of the estimated 250,000–500,000 plant species has been phytochemically investigated [49]. Medicinal plants are particularly rich in secondary metabolites with antimicrobial potential, and their essential oils have attracted increasing interest as possible alternatives or complements to conventional antibiotics in the context of multidrug resistance [50]. These oils are renewable, biodegradable, and often act through synergistic interactions among their constituents, which may reduce the likelihood of rapid resistance development. Their applications extend to human and veterinary medicine, pharmaceuticals, food preservation, and disinfection. However, despite these potential benefits, their therapeutic use remains limited by some constraints, including chemical variability under culture conditions, instability, and potential toxicity at high doses.

Despite the recognized benefits of AMF, their combined effects on the growth, mineral nutrition, primary metabolism, essential oil chemotype, and biological activity of *O. compactum* remain insufficiently characterized. In particular, an integrated assessment of these responses within the same experimental system is needed to determine whether AMF inoculation improves plant productivity and medicinal quality. We hypothesized that AMF inoculation would enhance plant biomass and mineral nutrition while reshaping primary metabolism, secondary metabolite accumulation, essential oil composition, and biological activity. Accordingly, this study aimed to compare AMF-inoculated and non-inoculated plants in terms of: (i) shoot and root biomass; (ii) mineral and trace-element composition; (iii) protein, sugar, lipid, polyphenol, and flavonoid contents; (iv) essential oil yield and chemical profile; and (v) antioxidant and antibacterial activities.

## 2. Materials and Methods

### 2.1. Experimental Design

An arbuscular mycorrhizal fungal (AMF) complex was isolated from maize roots using rhizosphere soil from Oregano after a 3-month trap culture. The trap culture technique was performed according to the method described by [51]. The AMF inoculum represented a native consortium propagated on maize (*Zea mays* L.) grown in sterilized substrate mixed with rhizospheric soil collected from natural populations of *O. compactum*. The fresh inoculum consisted of colonized root fragments, AMF spores, hyphae, and the associated growth substrate. The AMF inoculum was identified and characterized as described in our previous study [52] and contained approximately 1690 spores per 100 g. The inoculum was therefore applied as a composite propagule source rather than as a single AMF isolate. Oregano seeds were sown in peat substrate and maintained under controlled conditions for one month until the emergence and establishment of young seedlings. Mycorrhizal inoculation was performed by placing 100 g of fresh inoculum in direct contact with the roots of Oregano seedlings at the time of transplantation to polyethylene bags filled with a mixture of disinfected substrate and incubated at a temperature between 25° and 30 °C, a photoperiod of 14/10 h, and 70% relative humidity. The inoculum was placed directly below and in contact with the root system to facilitate root colonization. Two treatments were established: inoculated and non-inoculated plants, with 10 plants per treatment and 20 plants in total. To minimize treatment differences unrelated to viable AMF propagules, non-inoculated control plants received an equivalent quantity of autoclaved inoculum, providing comparable amounts of organic matter, root fragments, and mineral components. A microbial filtrate prepared from fresh inoculum and passed through filter paper was also applied to the control treatment to restore the associated non-mycorrhizal microbial community while excluding AMF spores and hyphal fragments. All pots contained the same sterilized growth substrate, and inoculated and non-inoculated plants were handled separately during transplantation and irrigation to minimize cross-contamination. The plants were arranged in a completely randomized design, irrigated daily, and grown for twelve months.

### 2.2. Assessment of Mycorrhizal Colonization

Mycorrhizal colonization of roots was evaluated following the method of [27]. Fine root samples were collected at harvest, thoroughly washed with tap water to remove adhering soil particles, and cut into 1 cm segments. Root fragments were cleared in 10% (*w*/*v*) KOH at 90 °C for 30–60 min, rinsed with distilled water, and acidified in 1% HCl for 10 min. Staining was performed using 0.05% trypan blue in lactoglycerol. After staining, roots were destained in lactoglycerol and mounted on slides for microscopic observation. Mycorrhizal parameters were quantified using the gridline intersect method under a compound microscope. The following indices were calculated: Frequency of mycorrhization (F%), representing the percentage of root fragments colonized; Intensity of colonization (M%), reflecting the extent of fungal structures within the root cortex; arbuscular abundance (A%), indicating the proportion of arbuscules; and vesicular abundance (V%), corresponding to storage structures. At least 30 root fragments per sample were examined to ensure statistical reliability.

### 2.3. Plant Material and Biomass Measurements

Aerial parts of Oregano cultivated with and without mycorrhizal inoculation at the flowering stage were obtained from seedlings cultivated under greenhouse conditions. The fresh biomass of each plant was recorded immediately after harvesting using a digital balance, and dry biomass was determined after drying the aerial parts at room temperature (≈25 °C) for one week. The dried material was then ground to 0.1–0.4 mm using a grinder and stored in clean bags until use in essential oil extraction.

### 2.4. Preparation and Digestion of Shoot and Root Samples

Shoots and roots of Oregano plants were separated and oven-dried at 70 °C for 48 h, after which the biomass was finely ground and ashed at 550 °C for 5 h. Two grams of the resulting powder were subsequently oxidized with 5 mL of H_2_O_2_ and heated in a sand bath to dryness. The residues were then digested with 5 mL of aqua regia, a mixture of concentrated nitric acid (HNO_3_) and hydrochloric acid (HCl) in a 1:3 ratio previously treated with H_2_O_2_. Mineralization was carried out by placing the samples in Teflon vessels on a heating plate and gradually increasing the temperature to approximately 200 °C for 2 h. The resulting digests were filtered through a 0.45 µm membrane and brought to a final volume of 10 mL using deionized water.

#### 2.4.1. Essential Mineral Nutrients and Potentially Toxic Trace Elements Measurements

The concentrations of mineral elements were determined in the filtrates of the different samples using an inductively coupled plasma emission spectrophotometer (ICPE-9000, Shimadzu, Japan). The analyzed elements were classified into two groups. Essential mineral nutrients included calcium (Ca), copper (Cu), iron (Fe), manganese (Mn), magnesium (Mg), phosphorus (P), potassium (K), sodium (Na), sulfur (S) and zinc (Zn). Potentially toxic trace elements comprised aluminum (Al), arsenic (As), barium (Ba), cadmium (Cd), chromium (Cr), nickel (Ni), strontium (Sr), uranium (U), and vanadium (V).

#### 2.4.2. Essential Oils Extraction

Extraction was carried out by hydrodistillation using a Clevenger-type apparatus [53]. Distillation was carried out with 60 g of plant powder in 1 L of distilled water for 3 h at 100 °C [54]. Oregano powder was mixed with distilled water in a heated flask topped with a graduated column, the ‘condenser’. The water vapor loaded with essential oil passes through the condenser; the hydrosol and essential oil are collected in a test tube, and the oil is recovered in an Eppendorf tube and kept at 4 °C in the dark.

#### 2.4.3. Chromatographic Analysis

Gas chromatography–mass spectrometry (GC–MS) was used to characterize the chemical composition of essential oils obtained from the dried aerial parts of AMF-inoculated and non-inoculated plants. Separation was achieved on a fused-silica capillary column HP-5MS (30 m × 0.25 mm i.d., 0.25 μm film thickness; Agilent Technologies; Santa Clara, CA, USA). Helium (purity ≥ 99.999%) was used as the carrier gas at a constant flow rate of 1.0 mL min^−1^. The injector temperature was maintained at 250 °C, and samples (1 μL) diluted in HPLC-grade hexane (1:100, *v*/*v*) were injected in split mode (split ratio 1:20). The oven temperature was programmed to increase from 50 °C (held for 3 min) to 250 °C at a rate of 4 °C min^−1^, with a final hold of 10 min. The mass spectrometer operated in electron impact (EI) ionization mode at 70 eV. The ion-source and transfer-line temperatures were maintained at 230 °C and 280 °C, respectively. Mass spectra were acquired over the m/z range of 40–500. Compound identification was based on the comparison of their mass spectra with those contained in the NIST 20 and Wiley spectral libraries, together with comparison of their calculated linear retention indices (LRIs) with published literature values. Linear retention indices were determined using a homologous series of *n*-alkanes (C8–C30) analyzed under identical chromatographic conditions according to the method of Van den Dool and Kratz. Only compounds showing a spectral similarity index ≥ 90% and retention indices consistent with published reference values were considered positively identified. Whenever available, the identification of the major constituents (carvacrol, thymol, p-cymene and γ-terpinene) was confirmed by comparison with authentic reference standards analyzed under the same experimental conditions. The relative percentage of each constituent was calculated by normalization of the GC peak areas without applying response-factor correction.

#### 2.4.4. Protein Determination

Protein content was determined using the Bradford method [55]. Bovine serum albumin standards ranging from 0 to 1 mg mL^−1^ were prepared in phosphate-buffered saline. Bradford reagent (3 mL) was added to 100 µL of each standard or plant sample extract. After incubation at room temperature for 15 min, the absorbance was measured at 595 nm. Protein concentrations were calculated from the bovine serum albumin calibration curve.

#### 2.4.5. Carbohydrate Content

The extracts were prepared by adding 0.1 g of powder to 10 mL of distilled water, then heating the mixture in a water bath at 90 °C for 30 min, centrifuging at 5000× *g* rpm, and recovering the supernatant. A total of 0.5 mL of 5% phenol was added to 1 mL of each Oregano extract used [56], after which 2.5 mL of sulfuric acid was poured onto the mixture and the whole mixture was left in a water bath at 30 °C for 20 min, followed by incubation for 10 min, and the absorbance was read at 492 nm. The total sugar concentrations were determined from a glucose calibration curve.

#### 2.4.6. Lipid Content

In total, 5 g of Oregano powder was used with 100 mL of chloroform/methanol (2/1, *v*/*v*), and the mixture was stirred for 20 min at room temperature. An aqueous solution of NaCl (0.9%) was added, and the mixture was centrifuged at 3000× *g* rpm for 10 min; the lower organic phase was recovered and distilled under reduced pressure using rotary evaporation. The lipid content was determined according to Equation and expressed as a percentage (%).

$$\%Lipids=\left(\frac{M2}{M1}\right)\times 100$$

where % Lipids is the lipid content, M1 is mass of the test sample, and M2 is lipid mass.

#### 2.4.7. Polyphenol Content

Total phenolic compounds contained in the essential oils of Oregano were quantified using the Folin–Ciocalteu reagent according to the method of [57]. In total, 0.2 mL of 3 essential oil solutions of mycorrhizal and non-mycorrhizal Oregano, freshly prepared by diluting the essential oils in 80% ethanol, and 0.2 mL of Folin–Ciocalteu reagent, were mixed vigorously with 1 mL of 15% sodium carbonate Na_2_CO_3_. The mixture was incubated for 2 h at room temperature, and the reading was taken against a gallic acid blank at 760 nm.

#### 2.4.8. Total Flavonoids Content

The total flavonoid content was determined by a colorimetric method [58]. A total of 1 mL of the plant extract was used, and 4 mL of distilled water was then added to the mixture, after which 0.3 mL of 5% NaNO_2_ solution was added. After 5 min, 0.3 mL of 10% AlCl_3_ was added. At the sixth minute, 2 mL of 1 M NaOH was added, and the final volume was brought to 10 mL with distilled water. The solution was thoroughly mixed, and the absorbance was measured at 510 nm using a spectrophotometer. Rutin was used as a standard, and the data were expressed as mg of rutin equivalent (RE/g DW).

#### 2.4.9. Antioxidant Activity and 2,2′-Diphenyl-1-Picrylhydrazyl (DPPH) Radical Scavenging Assay

The DPPH radical scavenging activity of the essential oil of mycorrhizal and non-mycorrhizal Oregano leaf powder was evaluated by the DPPH radical trapping test. The quantitative estimation of this activity was carried out according to previously described protocol. A 2 mL volume of the methanolic DPPH solution, prepared by solubilizing 2.4 mg of DPPH in 100 mL of methanol, was added to 100 µL of each mixture of mycorrhizal and non-mycorrhizal essential oils at different concentrations. The mixture was vortexed for 5 min, then incubated in the dark at room temperature for 30 min. The absorbance was then measured at 517 nm using a spectrophotometer against a blank and compared to a standard (ascorbic acid). The trapping activity was expressed as a percentage of inhibition (I%) calculated using the following equation:

$$I\%=\left(A\;blank-\frac{A\;sample}{A\;blank}\right)\times 100$$

The curves of the concentrations of essential oils and ascorbic acid as a function of the percentage inhibition of the DPPH radical were plotted, thereby allowing for determination of the IC_50_ index. The latter corresponds to the concentration of antioxidants necessary to reduce the initial concentration of the DPPH radical by 50%.

#### 2.4.10. Antibacterial Activity of Essential Oils from Mycorrhizal and Non-Mycorrhizal *O. compactum* Plants

The essential oil mixtures from mycorrhizal and non-mycorrhizal Oregano were evaluated for antibacterial activity against various bacterial strains (Gram-positive and Gram-negative). The following microbial strains were used: *Bacillus subtilis* (Gram+), *Staphylococcus aureus* (Gram+), *Micrococcus luteus* (Gram+), *Pseudomonas aeruginosa* (Gram−), and *Proteus mirabilis* (Gram−) (Table 1).

##### Inoculum and Bacterial Suspension Preparation

The antibacterial tests were carried out on young bacterial cultures in the exponential growth phase, aged 18 to 24 h, and 2 colonies of each isolated strain were subcultured and placed in 9 mL of physiological water, adjusted to a turbidity of 0.5 McFarland, and then incubated at 37 °C for 24 h.

##### Determination of Inhibition Zones

The antimicrobial activity of the essential oil mixtures from mycorrhizal and non-mycorrhizal *O. compactum* leaf powder was evaluated by the disk diffusion method [64]. Mueller–Hinton agar powder was dissolved in distilled water, and the mixture was autoclaved at 120 °C for 20 min. The agar was then poured aseptically into Petri dishes. Further, 6 mm paper disks were impregnated with 5 µL of the essential oil and placed in the Petri dishes inoculated with the bacterial suspensions. The antibiotic ciprofloxacin, at a concentration of 5 µg/disk, was used as a positive control. disks without samples were used as a negative control. The inhibition zones were compared with those of the controls after incubation at 37 °C for 24 h. The presence or absence of the absolute inhibition zone was used as a criterion to define the action of the essential oils (active or inactive). The tests were performed in triplicate.

##### Determination of Minimum Inhibitory Concentration in Liquid Medium

The minimum inhibitory concentration (MIC) of an essential oil is the smallest amount of that oil that prevents bacteria from multiplying. U-bottom microplates were used for MIC determination. A 96-well plate was used to determine MICs for the essential oils. In each well of the same row, 100 µL of Mueller–Hinton broth was introduced. Then, 100 µL of essential oil was added to the first well. A 1/2 dilution series was prepared directly in Mueller–Hinton broth, starting with the stock solution, which was placed in the first well. This dilution was carried out by transferring 100 µL from one well to the 9th well, then discarding 100 µL from the 9th well. Afterward, each well was inoculated with 10 µL of the microbial suspension at a concentration of 10^6^ cells/mL.

### 2.5. Statistical Analysis

Statistical analyses were performed using SPSS software (version 29.0). Data on protein, carbohydrate, lipid, total polyphenol, and total flavonoid contents, as well as IC_50_, inhibition zone diameter, and minimum inhibitory concentration values, were expressed as the mean ± standard deviation of three independent biological replicates per treatment, each represented by an individual plant. Normality and homogeneity of variance were verified before analysis. Differences among treatments were assessed by one-way ANOVA, followed by Tukey’s HS test for multiple comparisons when significant effects were detected. Statistical significance was set at *p* < 0.05. Principal component analysis (PCA) was performed to identify the main sources of variation among treatments and measured variables, and the first two principal components were visualized using a biplot. Pearson’s correlation analysis was used to assess the strength and direction of associations among the parameters studied, with significance set at *p* < 0.05.

## 3. Results

### 3.1. Composition and Diversity of the AMF Complex Used

The AMF consortium used in this study was overwhelmingly dominated by members of the order *Glomerales*, accounting for roughly 99% of the total community composition. Within this order, the families *Glomeraceae* and *Claroideoglomeraceae* accounted for the bulk of sequences, comprising 98% and 0.4%, respectively. A minor fraction (0.25%) belonged to the order *Paraglomerales*, which is represented exclusively by the family of *Paraglomeraceae*. At the genus level, *Glomus* was the most abundant, comprising 84% of sequences, followed by *Rhizophagus* (14.8%), with *Claroideoglomus* representing a small portion (0.4%). Rare genera such as *Sclerocystis* (0.1%) and *Paraglomus* (0.01%) were also detected, and approximately 0.69% of sequences could not be assigned to any known genus. Species-level analysis revealed that the AMF consortium included *Rhizophagus clarus*, *Rhizophagus intraradices*, *Paraglomus majewskii* and *Sclerocystis sinuosa*, in addition to multiple unidentified species affiliated with the genera *Paraglomus* and *Glomus*. These percentages represent relative sequence abundance and should not be interpreted as measurements of viable spore abundance, infective-propagule density, or the colonization capacity of the individual taxa. This composition illustrates a community dominated by well-characterized AMF taxa, yet retaining a fraction of unclassified diversity, highlighting both its ecological relevance and the potential for discovering novel fungal lineages within the consortium.

### 3.2. Plant Growth Performance of Inoculated and Non-Inoculated Origanum Compactum Seedlings

**Figure 1:**
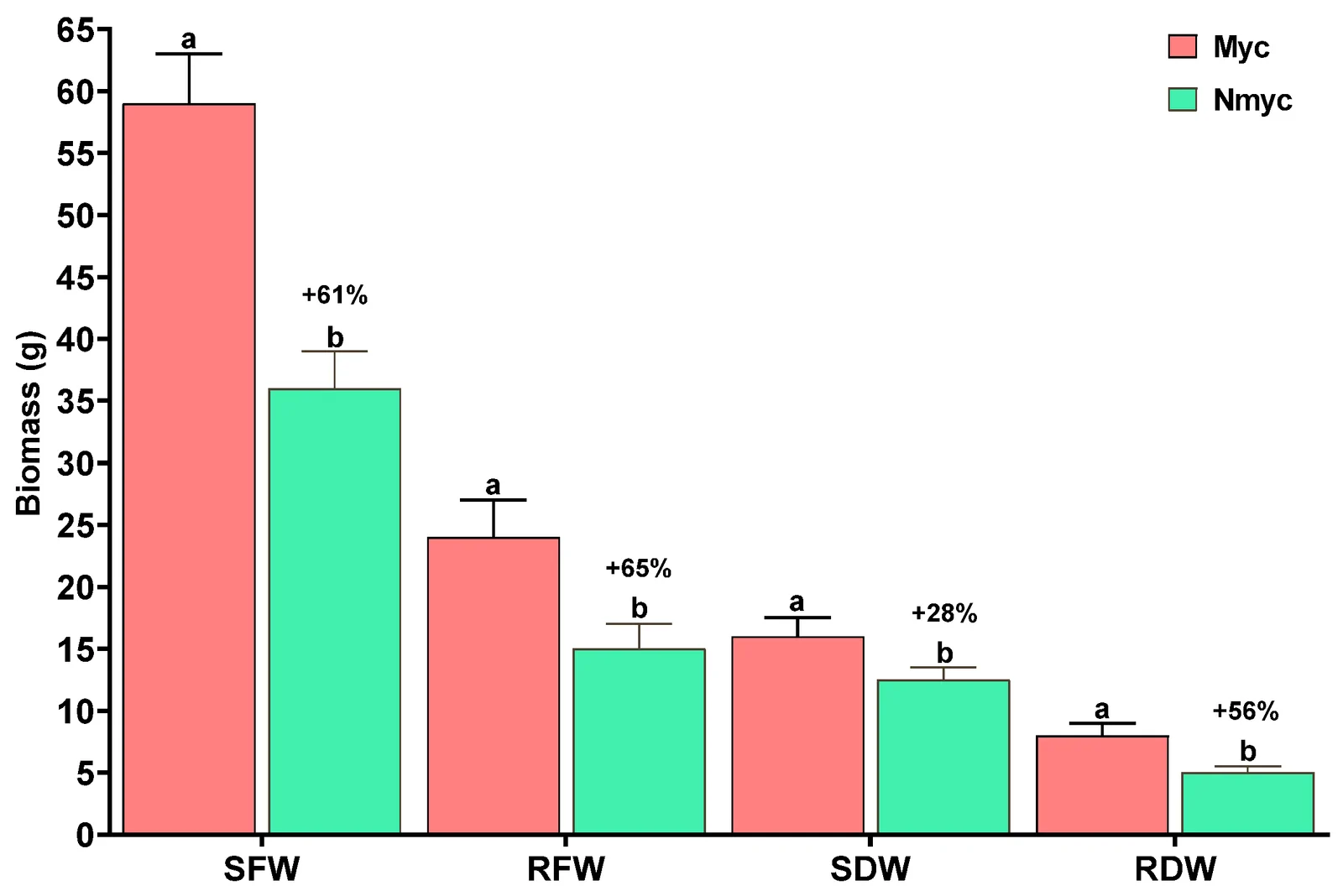
Effect of arbuscular mycorrhizal fungal (AMF) inoculation on the fresh and dry biomass of shoots and roots of *Origanum compactum*. Different letters indicate statistically significant differences among treatments according to Tukey’s HSD test (*p* < 0.05). Percent biomass increases induced by mycorrhizal inoculation are indicated for each biomass parameter in the figure. Nmyc, non-inoculated control plants; Myc, AMF-inoculated plants; SFW, shoot fresh weight; SDW, shoot dry weight; RFW, root fresh weight; RDW, root dry weight.

Arbuscular mycorrhizal inoculation significantly enhanced all growth parameters of *Origanum compactum* (Figure 1). The most pronounced effects were observed for fresh shoot and root biomass (*p* < 0.01), indicating improved water and nutrient acquisition. Post hoc Tukey’s test confirmed that mycorrhizal plants consistently exhibited higher biomass compared to non-mycorrhizal controls (*p* < 0.05). The substantial increases, particularly in root biomass, suggest improved belowground development, likely contributing to enhanced plant vigor and resource-uptake efficiency.

Mycorrhizal inoculation resulted in marked biomass gains, with increases of +61% and +65% in shoot and root fresh biomass, respectively. Dry biomass also improved significantly, particularly in roots (+56%), reflecting enhanced belowground carbon allocation. These results highlight the strong positive effect of mycorrhization on both biomass accumulation and partitioning, supporting its role in improving plant establishment under potentially limiting conditions.

### 3.3. Mycorrhizal Colonization Traits in Inoculated and Non-Inoculated Origanum compactum Seedlings

Mycorrhizal inoculation significantly increased all colonization parameters, with frequency (F%) reaching 75% and intensity (M%) 50%, compared to negligible levels in non-inoculated plants (Table 2). The presence of arbuscules (20%) and vesicles (30%) confirms the establishment of a functional symbiosis. These structures indicate active nutrient exchange and fungal storage, respectively, demonstrating an efficient and well-developed mycorrhizal association in inoculated plants.

### 3.4. Mineral Nutrition in Inoculated and Non-Inoculated Origanum compactum Seedlings

**Figure 2:**
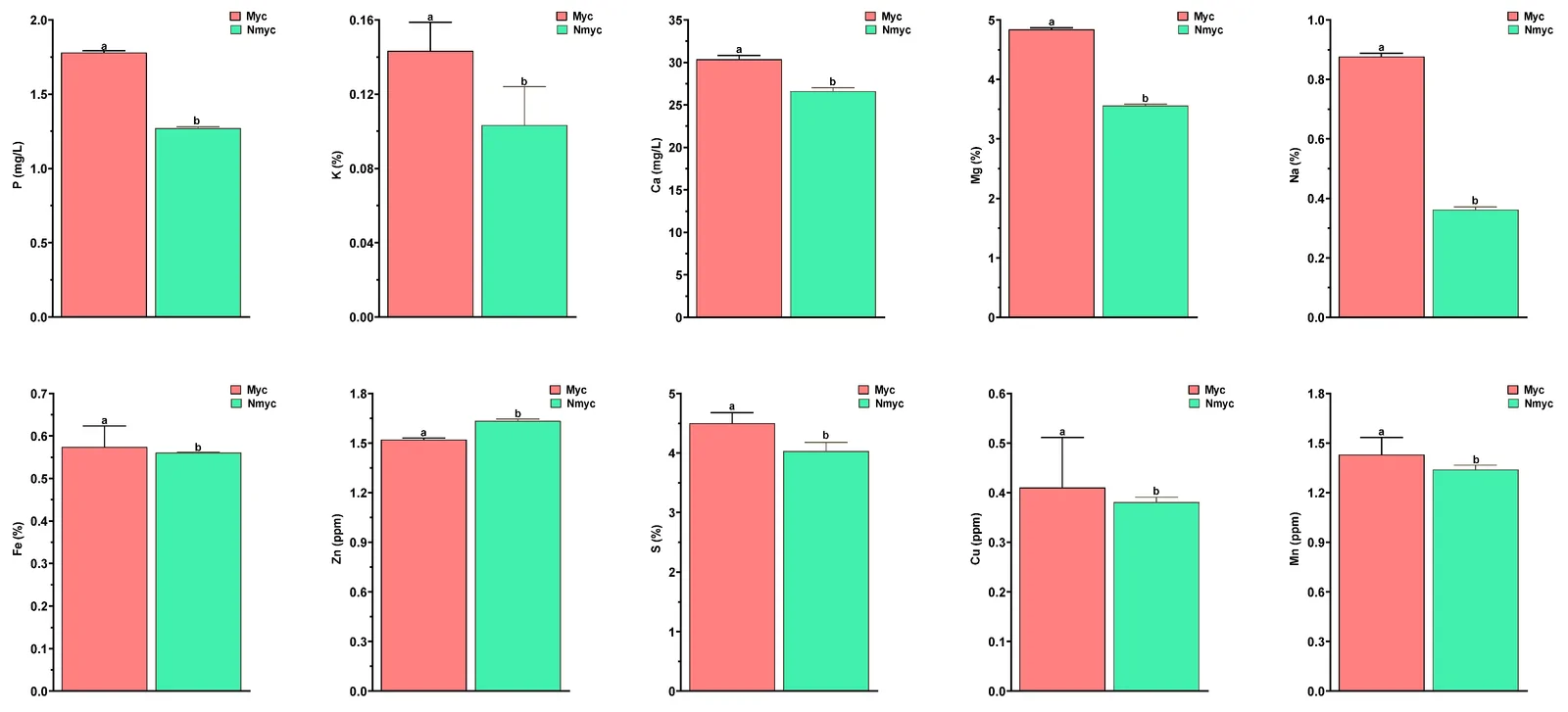
Effect of mycorrhization on essential mineral nutrients in *Origanum compactum* plants. Values are means ± SD (n = 3). Different letters indicate significant differences (Tukey HSD, *p* < 0.05). Ca = Calcium; Cu = Copper; Fe = Iron; Mn = Manganese; Mg = Magnesium; P = Phosphorus; K = Potassium; Na = Sodium; S = Sulfur; Zn = Zinc.

Mycorrhizal inoculation significantly improved the accumulation of most essential nutrients, particularly Mg, Na, Ca, and S, indicating enhanced mineral uptake efficiency. The strong increase in Mg (+~36%) suggests improved chlorophyll synthesis and photosynthetic capacity. Similarly, Zn showed a slight but significant decrease, possibly due to competitive uptake interactions. Overall, the results confirm that mycorrhization enhances nutrient acquisition and alters nutrient stoichiometry (Figure 2).

### 3.5. Potentially Toxic Elements in Inoculated and Non-Inoculated Origanum compactum Seedlings

**Figure 3:**
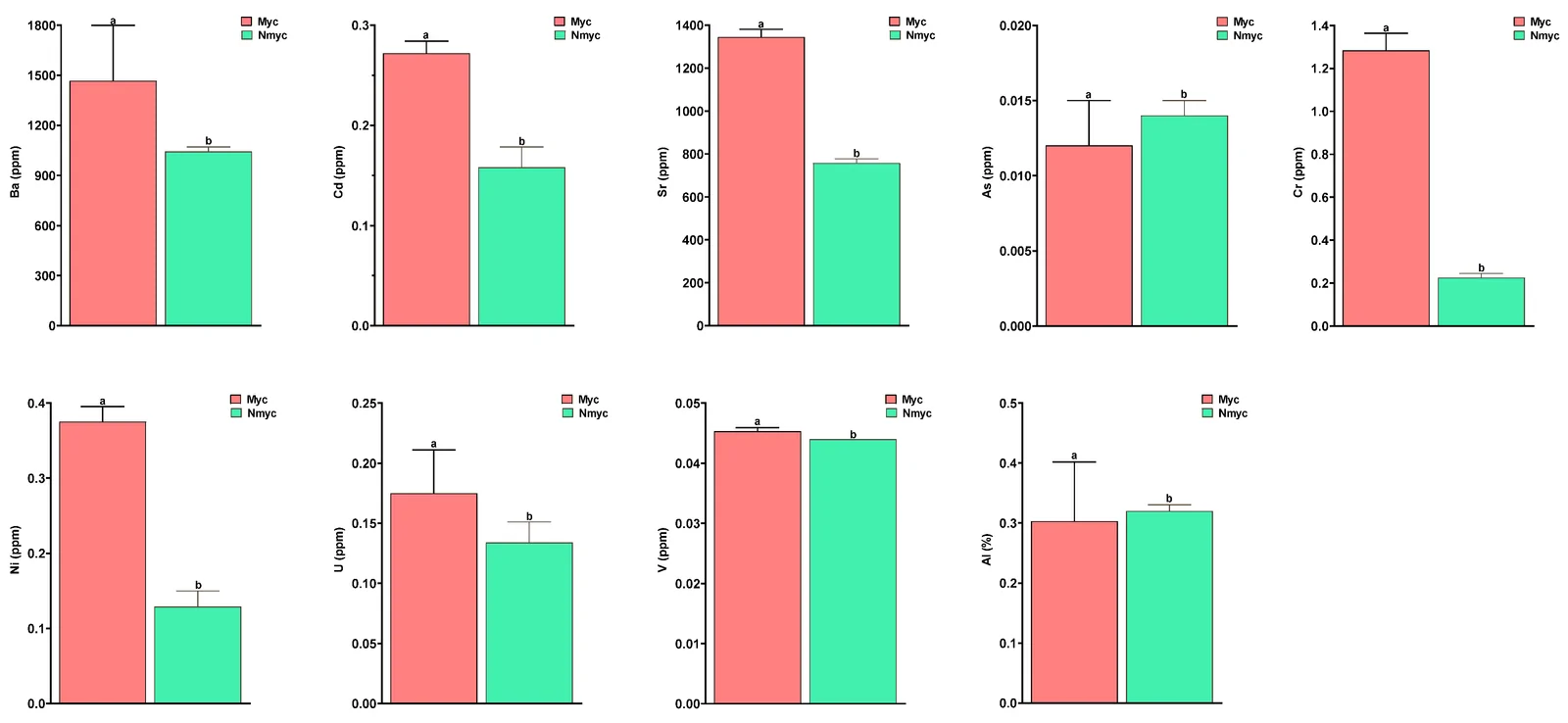
Effect of mycorrhization on trace and potentially toxic elements in *Origanum compactum* plants. Values are means ± SD (n = 3). Different letters indicate significant differences (Tukey HSD, *p* < 0.05). Al = Aluminum; As = Arsenic; Ba = Barium; Cd = Cadmium; Cr = Chromium; Ni = Nickel; Sr = Strontium; U = Uranium; V = Vanadium.

Mycorrhization significantly influenced the accumulation of trace and potentially toxic elements. Notably, a strong increase in Cr, Ni, Sr, and Cd indicates enhanced metal uptake or mobilization in the rhizosphere (Figure 3). The decrease in Al suggests a protective effect against aluminum toxicity. Slight reductions in As may reflect selective exclusion mechanisms. These results highlight a dual role of mycorrhizae, enhancing nutrient acquisition and increasing the uptake of certain trace elements. This suggests that *Origanum compactum* associated with mycorrhizae may exhibit phytoaccumulation potential, which could be advantageous for phytoremediation but requires careful monitoring in agricultural contexts. The symbiosis strongly alters plant mineral profiles and nutrient balance.

### 3.6. Quality Control of the Investigated O. compactum Essential Oils

Table 3 compared to non-mycorrhizal plants, those inoculated with mycorrhizae produced a higher essential oil output, distinguished by its pleasant fragrance and yellowish hue.

### 3.7. Analysis of the Essential Oil Extracted from Mycorrhizal and Non-Mycorrhizal O. compactum Plants

The qualitative and quantitative compositions of the EOs were determined using GC-MS (Table 4). Analysis of the essential oil composition showed that inoculation with AMF did not affect the nature of the compounds in the essential oil, but rather affected the abundance of the major compounds.

### 3.8. Proteins, Carbohydrates and Lipid Contents in Mycorrhizal and Non-Mycorrhizal Origanum compactum Leaves

The obtained results demonstrate that mycorrhizal inoculation influenced the primary metabolite content of Oregano plants in various ways. Mycorrhizal plants exhibited the highest protein content (1.21 mg/mL) compared to non-mycorrhizal plants (0.91 mg/mL), suggesting an evolution and improvement in protein synthesis linked to better nutrient assimilation facilitated by mycorrhizal symbiosis. Similarly, the carbohydrate concentration was significantly higher in Oregano mycorrhizal plants (100.37 mg/L), compared to non-mycorrhizal plants, which did not exceed 78.52 mg/L. The lipid content was slightly higher in non-mycorrhizal plants (1.76%) compared to mycorrhizal plants (1.64%) (Table 5).

### 3.9. Polyphenols and Flavonoids Content in Essential Oils of O. compactum with and without Mycorrhizal Inoculation

Polyphenols constitute a major class of bioactive compounds occurring in plants as secondary metabolites. The analysis showed that the essential oils extracted from mycorrhizal Oregano represented a significantly higher polyphenol content (102 mg GAE/g EO) than that emanating from non-mycorrhizal *O. compactum* (76.58 mg GAE/g EO) (Table 6).

The flavonoid content of the extracts was expressed as mg of rutin equivalent per gram of dry matter based on the calibration curve. The flavonoid contents recorded in mycorrhizal *O. compactum* were significantly higher, with a value of around 80.74 mg Eq rutin/g MS, than in the non-mycorrhizal plant, at 64.44 mg Eq rutin/g MS, an increase of about 25.3 (Table 6).

### 3.10. Antioxidant Activity of Essential Oils

The essential oil extracted from mycorrhizal Oregano has a lower IC_50_ value than that of non-mycorrhizal plants, indicating that the essential oil from the mycorrhizal plant possesses significantly greater antioxidant activity. Furthermore, this value was even slightly higher than that of the standard control, ascorbic acid, signifying a strong capacity for scavenging free radicals (Table 7).

### 3.11. Correlation of Phenolics and Flavonoids with Antioxidant Activity in Mycorrhizal and Non-Mycorrhizal O. compactum

Correlation analysis among phenolic compounds, flavonoids, and antioxidant activity revealed distinct patterns depending on the plants’ mycorrhizal status. In mycorrhizal plants, a moderate negative correlation was observed between total polyphenol content and DPPH IC_50_ values (r = −0.50), indicating that higher phenolic levels were associated with greater antioxidant activity. In contrast, this relationship was much stronger in non-mycorrhizal plants (r = −0.90), suggesting that antioxidant activity in the absence of symbiosis depends heavily on phenolic compounds. Regarding flavonoids, a positive correlation with IC_50_ values was observed in both cases, with a moderate correlation in mycorrhizal plants (r = +0.50) and an extremely strong correlation in non-mycorrhizal plants (r = +0.99). This suggests that flavonoids alone may not directly contribute to antioxidant efficiency and could even be associated with reduced activity, particularly in non-mycorrhizal conditions. Quantitatively, mycorrhizal plants exhibited higher levels of total phenolic compounds (102–106 mg GAE/g EO) and flavonoids (78–82 mg RE/g), along with lower IC_50_ values (0.006–0.008 mg/mL), reflecting a stronger antioxidant capacity compared to non-mycorrhizal plants. These results suggest that mycorrhizal symbiosis enhances antioxidant activity not only through increased phenolic accumulation but also via more complex metabolic interactions, likely involving synergistic effects among different classes of secondary metabolites (Table 8).

### 3.12. Antibacterial Activity

The antibacterial test results for mycorrhizal Oregano essential oil showed broad-spectrum activity against all tested bacterial strains. Inhibition zone diameters ranged from 42.5 to 57 mm, with the highest activity observed against *M. luteus* (57 mm), followed by *P. aeruginosa* (55 mm), *B. subtilis* (52 mm), *S. aureus* (49 mm), and *P. mirabilis* (42.5 mm) (Table 9). Non-mycorrhizal *O. compactum* essential oil also exhibited antibacterial activity against both Gram-positive and Gram-negative strains, although inhibition zones were consistently smaller, ranging from 35 to 46 mm. The greatest activity of the non-mycorrhizal oil was recorded against *B. subtilis* (46 mm) and *M. luteus* (45 mm), followed by *P. mirabilis* (38 mm), *S. aureus* (36 mm), and *P. aeruginosa* (35 mm). These results indicate that mycorrhizal inoculation enhanced the antibacterial activity of *O. compactum* essential oil across all tested bacterial strains.

These results indicate that mycorrhizal inoculation enhanced the antibacterial activity of *O. compactum* essential oil across all tested bacterial strains.

Ciprofloxacin (5 µg/disk) showed the highest overall antibacterial activity, producing inhibition zones of 57.5, 61, 82, 45, and 86 mm against *B. subtilis*, *M. luteus*, *P. aeruginosa*, *P. mirabilis*, and *S. aureus*, respectively. Nevertheless, the mycorrhizal essential oil exhibited an inhibition zone against *P. mirabilis* (42.5 mm) close to that produced by ciprofloxacin (45 mm), highlighting the comparatively strong activity of the mycorrhizal oil against this strain.

The minimum inhibitory concentrations (MICs) confirmed the antibacterial activity of *O. compactum* essential oil against all tested strains (Table 10). Mycorrhizal essential oil exhibited MIC values ranging from 0.156 to 0.625 mg/mL, with the greatest activity against *M. luteus* (0.156 mg/mL), followed by *B. subtilis* (0.312 mg/mL), *P. aeruginosa* (0.313 mg/mL), *S. aureus* (0.5 mg/mL), and *P. mirabilis* (0.625 mg/mL). In comparison, the non-mycorrhizal essential oil showed higher MIC values, ranging from 0.312 to 1.25 mg/mL. Mycorrhizal inoculation was therefore associated with enhanced antibacterial activity against *B. subtilis*, *M. luteus*, *P. aeruginosa*, and *S. aureus*, whereas both treatments showed the same MIC against *P. mirabilis* (0.625 mg/mL).

Ciprofloxacin exhibited lower MIC values against most strains (0.02–0.156 mg/mL), although its MIC against *M. luteus* (0.156 mg/mL) was identical to that of the mycorrhizal essential oil. Overall, these findings indicate that mycorrhizal inoculation enhanced the antibacterial potency of *O. compactum* essential oil against most of the tested bacteria.

### 3.13. Multivariate Trait Analysis in Mycorrhizal O. compactum

**Figure 4:**
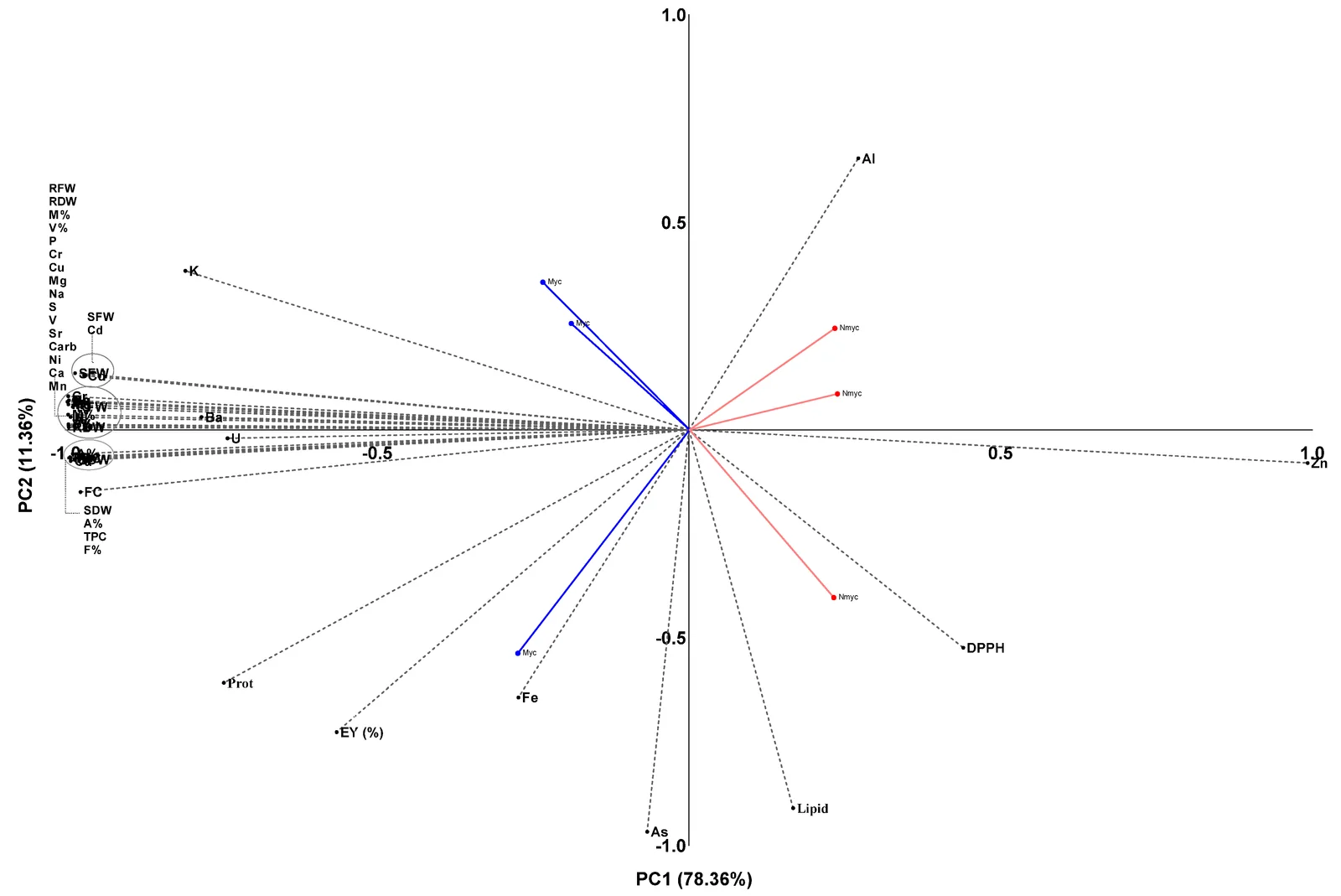
PCA biplot of Oregano traits under mycorrhizal treatment. The two colored point groups represent the distinct treatment categories, whereas the black dotted markers indicate the measured variables. The abbreviation Nmyc refers to non-mycorrhizal plants, while Myc denotes mycorrhizal plants. SFW = Shoot fresh weight; SDW = Shoot dry weight; RFW Root fresh weight; RDW = Root dry weight; F% = Frequency of mycorrhization; M% = Intensity of colonization; A% = Arbuscular abundance; V% = Vesicular abundance; Ca = Calcium; Cu = Copper; Fe = Iron; Mn = Manganese; Mg = Magnesium; P = Phosphorus; K = Potassium; Na = Sodium; S = Sulfur; Zn = Zinc; Al = Aluminum; As = Arsenic; Ba = Barium; Cd = Cadmium; Cr = Chromium; Ni = Nickel; Sr = Strontium; U = Uranium; V = Vanadium; EY (%) = Essential oil yield; Prot = Protein content; Carb = Carbohydrate content; Lip = Lipid content; TPC = Total phenolic concentration; FC = Flavonoids content; DPPH = DPPH radical scavenging activity.

Principal component analysis (PCA) revealed a clear multivariate differentiation between mycorrhizal (Myc) and non-mycorrhizal (Nmyc) *O. compactum* plants (Figure 4). The first two principal components explained 89.72% of the total variance, with PC1 and PC2 accounting for 78.36% and 11.36%, respectively. Treatment separation occurred predominantly along PC1, with Myc samples distributed on the negative side and Nmyc samples on the positive side. Most growth, mycorrhizal colonization, mineral, and phytochemical traits were associated with negative PC1 loadings. Among the mineral and trace elements, P, Cr, Cu, Mg, Na, S, V, Sr, Ni, Ca, and Mn clustered toward the negative PC1 region, together with Ba and U, while K showed a pronounced negative PC1 and positive PC2 loading. Fe was characterized by negative loadings on both components, whereas As was differentiated mainly by its strong negative PC2 loading. In contrast, Al and Zn were oriented toward positive PC1 and were more closely associated with the Nmyc treatment. The biochemical traits also showed distinct multivariate patterns: carbohydrate content (Carb) was associated with negative PC1 and positive PC2, whereas total phenolic content (TPC) and flavonoid content (FC) were strongly associated with negative PC1 and moderately negative PC2. Protein content and essential oil yield were positioned in the negative PC1/negative PC2 region, while lipid content and DPPH activity were associated with positive PC1 and negative PC2. Collectively, these patterns indicate that mycorrhizal inoculation was associated with coordinated changes in plant growth, colonization characteristics, mineral composition, and phytochemical traits, whereas the non-mycorrhizal treatment was characterized by a contrasting multivariate profile.

**Figure 5:**
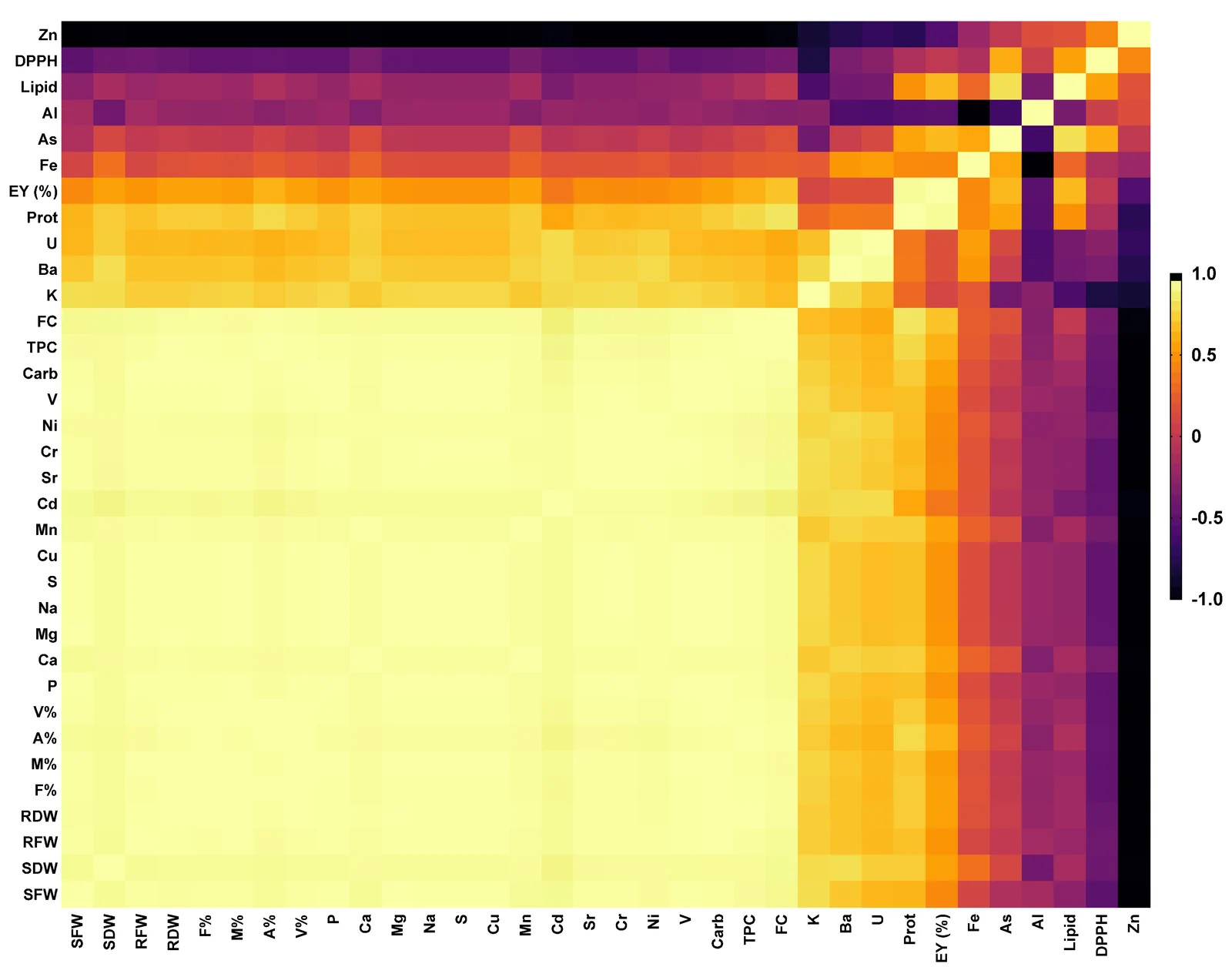
Heatmap visualization of Pearson correlations among studied variables. Box color intensity reflects the strength and direction of the Pearson correlation coefficient between each pair of variables. The heatmap legend displays correlation coefficients ranging from −1 to +1. SFW = Shoot fresh weight; SDW = Shoot dry weight; RFW Root fresh weight; RDW = Root dry weight; F% = Frequency of mycorrhization; M% = Intensity of colonization; A% = Arbuscular abundance; V% = Vesicular abundance; Ca = Calcium; Cu = Copper; Fe = Iron; Mn = Manganese; Mg = Magnesium; P = Phosphorus; K = Potassium; Na = Sodium; S = Sulfur; Zn = Zinc; Al = Aluminum; As = Arsenic; Ba = Barium; Cd = Cadmium; Cr = Chromium; Ni = Nickel; Sr = Strontium; U = Uranium; V = Vanadium; EY (%) = Essential oil yield; Prot = Protein content; Carb = Carbohydrate content; Lip = Lipid content; TPC = Total phenolic concentration; FC = Flavonoids content; DPPH = DPPH radical scavenging activity.

The Pearson correlation heatmap corroborated the PCA structure, revealing strong positive relationships among growth parameters, yield components, carbohydrates, phenolics, flavonoids, and multiple mineral elements (Figure 5). Conversely, Zn, Al, lipid content and DPPH activity variables showed weak or negative associations with the main growth–phytochemical trait cluster. Collectively, these findings indicate that mycorrhizal colonization substantially reorganized the physiological and biochemical performance of Oregano plantlets, with Myc plantlets exhibiting enhanced growth, nutrient status, and phytochemical potential compared with Nmyc plantlets.

## 4. Discussion

The present results clearly demonstrate that arbuscular mycorrhizal inoculation substantially alters biomass, mineral nutrition, and trace-element accumulation in *Origanum compactum*. The significant increases in essential nutrients, particularly Mg, Na, Ca, and S, indicate enhanced nutrient-use efficiency mediated by the mycorrhizal symbiosis. The marked rise in Mg content is especially noteworthy, as Mg is directly associated with chlorophyll synthesis and photosynthetic performance, which may partly explain the observed biomass improvements. Conversely, the slight but significant decrease in Zn suggests competitive interactions or dilution effects resulting from enhanced growth. In parallel, mycorrhizal inoculation also markedly affected the accumulation of trace and potentially toxic elements. In particular, the increases in Cr, Ni, Sr, and Cd suggest enhanced mobilization and uptake of these elements from the rhizosphere. Although such a response may be advantageous in phytoremediation contexts, it cannot be regarded as unequivocally beneficial when *O. compactum* is intended for culinary, medicinal, or phytopharmaceutical use. Excessive accumulation of Cr, Ni, and especially Cd may compromise the safety and medicinal quality of the harvested biomass, even when plant growth and bioactive properties are improved. Potentially toxic elements may also induce oxidative stress, interfere with nutrient homeostasis, and modify secondary-metabolite pathways, thereby affecting the chemical composition and biological activity of the plant. Conversely, the reduced concentrations of Al and As may indicate selective exclusion, immobilization in the rhizosphere, or mycorrhiza-mediated detoxification mechanisms. These contrasting responses confirm that AMF effects are element-specific and depend on factors such as fungal identity, soil properties, metal bioavailability, and plant genotype. Therefore, the observed increase in mineral accumulation should be interpreted as a dual response: AMF inoculation improved the nutritional status and growth of *O. compactum*, but it also increased the accumulation of several potentially harmful elements.

On the other hand, to improve the economic value of *Origanum compactum* essential oil, it would be imperative to increase the concentration of its bioactive compounds. The results obtained in this study demonstrate the role of mycorrhizal inoculation in improving the nutritional quality of the plant and its essential oil. These findings are in line with several previous works that reported the positive effect of AMF in biochemical composition of the essential oil on *Salvia officinalis* [65], *Thymus vulgaris* [66], *Dracocephalum moldavica* [67], *Rosmarinus officinalis* [68], *Salvia rosmarinus* [69], and *Lavandula angustifolia* [70]. More specifically, [71] reported that *Funneliformis mosseae* inoculation enhanced biomass, nutrient uptake, and essential oil yield in *Origanum onites* under drought conditions. This closely related evidence supports the present observation that mycorrhizal symbiosis can improve both nutritional status and essential oil production in *O. compactum*. In fact, the quality of the essential oil is indirectly linked to the phytohormones produced by the plant, which regulate growth and modulate the synthesis of secondary metabolites [72]. These phytohormones regulate the expression of genes encoding enzymes in the methylerythritol phosphate (MEP) and mevalonate (MVA) pathways in plastids and the cytosol, such as terpene synthase. They also have specific effects on the glandular hairs responsible for the secretion of essential oils [73]. In many aromatic and medicinal plants, the increase in the number of glandular trichomes directly increases terpenoid concentration [74]. Regarding the chemical composition of essential oils, a significant difference was observed between mycorrhizal and non-mycorrhizal plants. The concentrations of major compounds such as carvacrol and thymol were significantly affected. This improvement indicated more intense metabolic activity within the tissues of mycorrhizal plants. This was attributed to the ability of arbuscular mycorrhizal fungi to maximize the absorption of nutrients such as phosphorus and nitrogen, which are essential for amino acid synthesis and, therefore, protein synthesis. The increased protein content thus reflects improved metabolism and remarkable natural vitality in mycorrhizal *O. compactum*. This concept has been reported in several studies, where mycorrhization stimulated protein synthesis, leading to the growth of aromatic and medicinal plants [75]. As reported by [76], phosphorus and nitrogen are two essential nutrients for plant growth. AMF promote the absorption of inorganic phosphorus (Pi) and nitrogen (N) from the soil, which in turn ensures and maintains mycorrhizal symbiosis with the plant. While inorganic phosphorus is not a direct component of plant proteins, it is essential to their synthesis and function. Plants absorb inorganic phosphorus from the soil through their roots, which are extended by mycorrhizal hyphae to optimize uptake, and store it as an energy source to build their cell structures and membranes. It is also used in the production of plant proteins [77]. Arbuscular mycorrhizal fungi improve nitrogen nutrition in plants, which in turn promotes protein synthesis. Mycorrhizal symbiosis expands the root surface area by forming numerous underground channels that increase nutrient exchange with the fungi. This network of filaments absorbs nutrients from the soil, including nitrogen in the form of ammonium, and transports them to plant tissues via its extensive mycelium. The absorbed nitrogen is the key element for protein synthesis, which explains the high protein content of mycorrhizal plants. This is consistent with results from other studies conducted on *Trigonella foenum-graecum* L. [78].

In the present study, the colonization of the plant roots by the AMF significantly increased the total sugar content in the aerial part, which is in agreement with several studies carried out on *Citrus* [79]; however, in others, such as trifoliate orange mycorrhization, it altered the metabolism related to sugar production [80].

The difference between mycorrhizal and non-mycorrhizal plants indicated a stimulation of photosynthetic activity and increased sugar production. These sugars not only contribute to energy reserves but also serve as substrates for the biosynthesis of secondary metabolites, which constitute the main components of essential oils, and as substrates for the mycorrhizal fungus to support its respiratory activity [81]. The increased carbohydrate content observed in mycorrhizal plants may contribute not only to growth but also to physiological adjustment under stress. Reference [82] similarly reported that mycorrhizal symbiosis enhanced soluble sugar accumulation and supported physiological and antioxidant responses in *Origanum majorana* under drought stress, highlighting the role of carbohydrates in stress acclimation. Sugars also contribute to the stabilization of cellular structures and macromolecules under adverse conditions [83]. In the present study, the concurrent increases in carbohydrate and protein contents suggest that mycorrhization modulated primary metabolism in *O. compactum*. Such changes may influence secondary metabolism by altering the availability of carbon- and nitrogen-derived precursors required for the biosynthesis of terpenoid and other aromatic compounds that determine essential oil composition and quality [84].

The lipid content of non-mycorrhizal *O. compactum* is slightly higher than that observed in the mycorrhizal plant; this slight but significant difference reflects metabolic changes resulting from mycorrhizal symbiosis. This transfer causes a significant shift in carbon from the plant to the fungus as lipid precursors, since non-mycorrhizal plants do not carry out this transfer and therefore retain their share of lipids [85]. Lipids can be mobilized as needed across different parts of the plant and used as an energy source by the symbiotic mycorrhizal fungus to support its growth and development [45]. This can be explained by the fact that in the absence of mycorrhization, the plant may be under mild nutritional stress; it accumulates more lipids as a form of energy storage to meet its needs. Roots in symbiosis with the arbuscular mycorrhizal fungus develop structures called arbuscules and vesicles, which can lead to or promote a more dynamic use of lipids and thus a reduction in their content in the plant tissues of mycorrhizal plants [86].

The polyphenols in essential oils offer several benefits to human health. They can improve cardiovascular health, prevent autoimmune diseases such as diabetes, and are also known for their skin-repairing properties [87]. The polyphenols contained in essential oils, such as eugenol and bis-eugenol from clove, offer several benefits for human health; they are known for their anti-inflammatory and antioxidant properties [88] and anticancer effects [89]. The significant difference observed in our study indicated that mycorrhization positively influences the formation of secondary metabolites, particularly phenolic compounds. These results are consistent with several studies demonstrating that association with a mycorrhizal fungus enhances the synthesis of secondary metabolites, namely phenols, in a wide range of aromatic and medicinal plants, whether in plant extracts or essential oils [90]. Polyphenols play a major role in the interaction between arbuscular mycorrhizal fungi and plants, specifically in signaling [91]. Plant roots secrete polyphenols such as quercetin and quercitrin to promote contact between fungal propagules and roots [92]. The increase in primary and secondary metabolite levels in response to mycorrhization is consistent with several studies [93,94]. They also help strengthen plants’ self-defense mechanisms, making them more resistant to diseases and pests. This group of metabolites is essential for plant survival in the face of environmental stresses [87], whether from biotic factors (nematodes, herbivores) or abiotic factors (drought, UV radiation, temperature fluctuations, soil salinity).

In reality, the mycorrhizal association optimizes the assimilation of essential nutrients, which facilitates the biosynthesis of precursors of secondary metabolism, especially aromatic amino acids (phenylalanine and tyrosine), which are the basis of several phenols contained in essential oils of the genus *Origanum*. The increase in phenolic compounds observed in mycorrhizal *O. compactum* plants can be explained by the activation of the antioxidant system mediated by mycorrhizal symbiosis. Furthermore, several studies have demonstrated that AMF can induce moderate oxidative stress in the host, enhancing the synthesis of phenols that play a protective role in the plant. The higher flavonoid contents observed in mycorrhizal Oregano were explained by the fact that arbuscular mycorrhizal fungi stimulate the biosynthesis of these compounds and promote their accumulation in the plant tissues. These findings are consistent with those reported by [95], highlighting that multiple physiological mechanisms govern this process. Flavonoids are phenolic compounds that play several roles in plant–fungus interactions. They are chemical signals that act before mycorrhization occurs, facilitating recognition and contact between the fungus and plant roots. The plant roots release these chemical compounds into the soil to stimulate the development and branching of mycelial hyphae, as well as the synthesis of fungal spores for the propagation of arbuscular mycorrhizal fungi in the rhizosphere [96]. Flavonoids were not only chemical attractants but also provided a natural way to fight against several pathogenic fungi [97] and microbes [98]. They were also known for their anthelmintic [99], antioxidant, and anti-inflammatory properties [100]. Distinct metabolic behaviors between mycorrhizal and non-mycorrhizal plants in antioxidant activity were recorded. Under non-mycorrhizal conditions, phenolic compounds were the main contributors to radical-scavenging capacity, whereas flavonoids played a less effective role in antioxidant activity. In contrast, mycorrhizal plants exhibited weaker correlations for both polyphenols and flavonoids, suggesting that a broader range of metabolites influences antioxidant activity. This supports the hypothesis that mycorrhization enhances metabolic diversity and promotes the involvement of other bioactive compounds, such as terpenoids, in antioxidant mechanisms.

The antimicrobial activity tests on the essential oils obtained showed considerable efficacy against a broad spectrum of Gram-positive and Gram-negative pathogenic bacteria. The values obtained indicated that the essential oil of the mycorrhizal plant exhibited significantly higher antimicrobial activity against both Gram-positive and Gram-negative bacteria. This strong antimicrobial potential was evident from large inhibition zone diameters and low MIC values, even though the essential oils were diluted to 2 mg/mL, as the initial concentrations completely halted bacterial growth at the MIC level. This underscores the role of mycorrhization in enhancing the plant’s ability to produce secondary metabolites with strong antimicrobial potential. Reference [101] reported significant antimicrobial activity of *Ocimum basilicum* essential oil against several Gram-negative and Gram-positive strains. This observed improvement was due to several processes; arbuscular mycorrhizal symbiosis stimulates phenolic, terpenic and flavonoid compounds, particularly carvacrol and thymol, which were responsible for antimicrobial activity [102], thanks to the stimulation of the absorption of compounds such as phosphorus and nitrogen among many others [103], which promotes the biosynthesis of aromatic compounds. Several studies support these results. Reference [104] reported improvements in yield and nutritional quality in *Thymus daenensis*, *Thymus vulgaris* and *Ocimum basilicum* [105]. In summary, these results mean that inoculation with arbuscular mycorrhizal fungi presents an ecological strategy for optimizing the quality of essential oils from aromatic and medicinal plants, specifically in the case of *O. compactum*. Carvacrol and thymol exert their antimicrobial action through several mechanisms: they alter bacterial membrane permeability, leading to the leakage of ions and cell constituents; they denature membrane proteins, impacting internal metabolism; or they disrupt the nutrient transport gradient, all of which cause cell death or reduced bacterial growth. That makes mycorrhizal Oregano essential oil usable in several agri-food and pharmaceutical fields, thanks to its bactericidal biochemical compounds, mainly carvacrol and thymol. It can extend the shelf life of food and replace synthetic preservatives. Essential oils can also be used in food packaging to release molecules slowly onto the food surface for preservation [106]. Oregano essential oil may represent a promising and natural alternative to synthetic antibiotics, especially regarding bacterial resistance, thus opening up avenues for new therapeutic approaches.

The DPPH method was used to measure the antioxidant power of mycorrhizal and non-mycorrhizal *O. compactum* essential oils, compared with a reference antioxidant, ascorbic acid. This difference indicated that the lower the IC_50_ value, the higher the antioxidant potential of the substance, since a small amount of the essential oil was sufficient to scavenge 50% of DPPH free radicals. Therefore, mycorrhizal Oregano plants exhibit the strongest free radical scavenging activity, while non-mycorrhizal ones and ascorbic acid show significantly lower values. This clear difference was explained by the fact that mycorrhization stimulates the production of secondary metabolites in the essential oil, especially carvacrol and thymol. These antioxidant compounds can neutralize free radicals by donating a hydrogen atom or an electron, helping stabilize them and thus reducing oxidative stress. These results are consistent with other studies reporting that the essential oils of mycorrhizal plants exhibit the best antioxidant activity [107,108]. Zhao et al. [72] similarly concluded that AMF can enhance secondary-metabolite production directly through increased plant biomass and indirectly through stimulation of secondary-metabolite biosynthetic pathways. The strong antioxidant capacity of mycorrhizal Oregano essential oil suggests that mycorrhizal plants may be a promising, natural source of antioxidants. This finding highlights the use of natural, biodegradable antioxidants in the food, cosmetic, and pharmaceutical industries to replace synthetic antioxidants, which have long-term side effects in the human body. Therefore, the results of this study support promoting the use of essential oils from mycorrhizal plants as an alternative to artificial antioxidants, given that carvacrol and thymol offer several advantages. Carvacrol has been reported to be highly beneficial for the respiratory system and is also known for its antioxidant, antimicrobial, anti-inflammatory, and digestive properties, which make it suitable for inclusion in poultry feed [109]. This could not only support improvements in the quality and, especially, the stability of food and pharmaceutical products, but also, above all, optimize and provide protection against diseases related to oxidative stress. At the same time antimicrobial and antioxidant, *O. compactum* essential oil remains a first choice for use in the food industry as a natural biological preservative, to both inhibit microbial proliferation and prevent lipid oxidation and thus prevent food from going rancid while maintaining nutritional quality, or even to integrate it into antiseptic formulations such as ointments, shampoos, antiaging creams and disinfectants, with safety measures defined for each product category and each age group.

## 5. Conclusions

AMF inoculation markedly enhanced mineral nutrition and growth in Oregano (*Origanum compactum*), notably improving Mg, Ca, and S uptake. This benefit was accompanied by increased accumulation of potentially toxic trace metals such as Cr, Ni, and Cd, indicating a trade-off between nutrient acquisition and metal uptake, whereas reductions in Al and As suggest selective regulatory mechanisms within the symbiosis. Overall, Oregano exhibited an AMF-induced pattern of enhanced nutrient acquisition accompanied by limited selectivity for certain trace elements. Although this response may be relevant to phytoremediation, the accumulation of potentially toxic elements requires caution, particularly when the plant is cultivated for medicinal, culinary, or commercial purposes in metal-contaminated environments.

AMF inoculation also improved the essential oil (EO) yield and modified its chemical profile, including the relative abundance and detectability of several putatively annotated compounds, particularly carvacrol and thymol, as well as nutritional and functional traits, including protein, carbohydrate, polyphenol, and flavonoid contents. Some EO constituents were detected only in one treatment, whereas others decreased below the analytical detection limit in the other, indicating that AMF inoculation reshaped both the qualitative and quantitative volatile profiles. These enhancements may be associated with altered nutrient acquisition and the stimulation of primary and secondary metabolism; however, the underlying metabolic and defense mechanisms were not directly investigated. Consequently, AMF inoculation represents a promising sustainable biostimulant approach to increase the therapeutic and commercial value of Oregano. Future research should explore field-scale implementation, microbial consortia interactions, and the molecular basis of metabolite enrichment to advance eco-friendly production.

## Figures and Tables

**Table 1:** Characteristics of the bacterial strains.

Species	Nuisance–Effect
*Bacillus subtilis*	Improved digestive health through the efficient breakdown and assimilation of nutrients [59]
*Micrococcus luteus*	Tissue inflammation, septicemia [60]
*Proteus mirabilis*	Urinary infection [61]
*Pseudomonas aeruginosa*	Lung, urinary, skin, blood and ear infections [62]
*Staphylococcus aureus*	Infection of the bloodstream, respiratory tract and skin [63]

**Table 2:** Mycorrhizal colonization traits in *Origanum compactum* seedlings after 12 months of growth.

Mycorrhizal Colonization Traits	Nmyc	Myc
Frequency (F%)	0 ^b^	75 ^a^
Intensity (M%)	0 ^b^	50 ^a^
Arbuscules (A%)	0 ^b^	20 ^a^
Vesicles (V%)	0 ^b^	30 ^a^

Values followed by different letters differ significantly at *p* < 0.05.

**Table 3:** Mycorrhizal and non-mycorrhizal essential oil organoleptic characteristics.

Plant	Yield (%)	Color	Odor	Flavor	Solubility
Mycorrhizal plants	1.99 ± 0.02 ^a^	Dark Yellow	Strong aroma	Spicy	Fat soluble
Non-mycorrhizal plants	1.6 ± 0.01 ^b^	Whitish	Less strong aroma	Spicy	

Means followed by different letters are significantly different according to Tukey’s HSD test (*p* < 0.05).

**Table 4:** Chemical composition of essential oils extracted from Mycorrhizal and non-Mycorrhizal *O. compactum* plants cultivated under greenhouse conditions.

Product	Compounds	Mycorrhizal Plants		Non-Mycorrhizal Plants	
		Retention Time	Area	Retention Time	Area
1	α-Thujene	7.68	1.14	-	-
2	α-pinene	7.89	0.86	-	-
3	1-octen-3-ol	9.17	0.58	9.17	1.11
4	3-octanone	9.33	0.78	-	-
5	β-myrcene	9.49	1.1	-	-
6	α-Terpinene	-	-	10.33	2.17
7	4-Carene	10.33	1.7	-	-
8	p-Cymene	10.57	16.39	10.55	16.41
9	γ-terpinene	11.59	9.03	11.58	8.17
10	4-thujanol	11.92	1.17	-	-
11	linalool	12.79	2.32	-	-
12	Borneol	-	-	14.97	2.41
13	Terpinen-4-ol	15.23	1.56	15.23	1.61
14	α-Terpineol	15.74	3.69	-	-
15	Isothymol methyl ether	16.85	0.5	16.86	16.68
16	Thymol	18.3	2.08	18.26	2.29
17	Carvacrol	18.73	38.88	18.56	26.65
18	Caryophyllene	21.85	9.79	21.84	9.26
19	Alloaromadendrene	-	-	22.31	1.83
20	α-humulene	22.74	0.86	22.74	0.94
21	Viridiflorene	-	-	23.62	1.77
22	β-Bisabolene	-	-	23.96	2.41
23	iso-Caryophyllene	23.96	0.55	-	-
24	δ -cadinene	24.25	0.5	-	-
25	Spathulenol	-	-	25.88	1.72
26	Caryophyllene oxide	26.07	2.03	26.06	2.43
27	ˠ-clausenane	31	0.47	-	-
28	Rosefuran	34.54	1.51		
29	2,2′-methylenebis [6-(1,1-dimethylethyl)-4-methyl]- phenol	43.51	0.69	43.51	1.02
	Total		98.18		98.88

**Table 5:** Protein, carbohydrate and lipid content in leaf powder of mycorrhizal and non-mycorrhizal *O. compactum*.

Primary Metabolites	Mycorrhizal Plants	Non-Mycorrhizal Plants
Proteins (mg/mL)	1.21 ^a^	0.91 ^b^
Carbohydrates (mg/L)	100.37 ^a^	78.52 ^b^
Lipids (%)	1.64 ^a^	1.76 ^a^

Means followed by the same letter within a row are not significantly different according to Tukey’s HSD test (*p* < 0.05).

**Table 6:** Total phenolic and flavonoid content of essential oils from mycorrhizal and non-mycorrhizal *O. compactum* plants.

Essential Oil Origin	Total Amount of Phenolic Compounds (mg EAG/g EO)	Flavonoid Content (mg Eq Rutin/g MS)
Mycorrhizal plants	102 ^a^	80.74 ^a^
Non-Mycorrhizal plants	76.58 ^b^	64.44 ^b^

Means followed by the same letter within a column are not significantly different according to Tukey’s HSD test (*p* < 0.05).

**Table 7:** Antioxidant activity (IC_50_ mg/mL) of the essential oils extracted from Mycorrhizal and non-Mycorrhizal *O. compactum* plants.

	Mycorrhizal Plants	Non-Mycorrhizal Plants	Ascorbic Acid
**DPPH (** **IC50 mg/mL)**	0.007 ^b^	0.012 ^a^	0.013 ^a^

Means followed by the same letter within a row are not significantly different according to Tukey’s HSD test (*p* < 0.05).

**Table 8:** Correlation of polyphenols and flavonoids with DPPH antioxidant activity in mycorrhizal and non-mycorrhizal *O. compactum*.

Analyzed Parameter	Mycorrhizal Plants	Non-Mycorrhizal Plants
Polyphenols vs. IC_50_	−0.50	−0.90
Flavonoids vs. IC_50_	+0.50	+0.99
Interpretation	Moderate	High dependency

**Table 9:** Antibacterial activity of essential oils of mycorrhizal and non-mycorrhizal *O. compactum*.

Sample	Diameter of Inhibition Zone (mm)				
	*B. subtilis*	*M. luteus*	*P. aeruginosa*	*P. mirabilis*	*S. aureus*
Mycorrhizal *O. compactum*	52 ^b^	57 ^b^	55 ^b^	42.5 ^b^	49 ^b^
Non-mycorrhizal *O. compactum*	46 ^c^	45 ^c^	35 ^c^	38 ^c^	36 ^c^
Antibiotic (ciprofloxacin, 5 µg/disk)	57.5 ^a^	61 ^a^	82 ^a^	45 ^a^	86 ^a^

Means followed by the same letter within a column are not significantly different according to Tukey’s HSD test (*p* < 0.05).

**Table 10:** Minimum inhibitory concentration (MIC) values of essential oils from mycorrhizal and non-mycorrhizal *O. compactum*.

Sample	MICs (mg/mL)				
	*B. subtilis*	*M. luteus*	*P. aeruginosa*	*P. mirabilis*	*S. aureus*
Mycorrhizal plants	0.312 ^b^	0.156 ^b^	0.313 ^b^	0.625 ^a^	0.5 ^b^
Non-mycorrhizal plants	0.625 ^a^	0.312 ^a^	1.25 ^a^	0.625 ^a^	1.25 ^a^
Antibiotic (ciprofloxacin, 5 µg/disk)	0.04 ^c^	0.156 ^b^	0.04 ^c^	0.02 ^b^	0.04 ^c^

Means followed by the same letter within a column are not significantly different according to Tukey’s HSD test (*p* < 0.05).

## Data Availability

The datasets generated and/or analyzed during the current study are provided in the manuscript.
